# Book Reviews

**Published:** 1825-10

**Authors:** 


### CRITICAL ANALYSIS

OF

### ENGLISH AND FOREIGN LITERATURE,

RELATIVE TO THE VARIOUS BRANCHES OF

### Medical Science.

Quæ laudanda forent, et quæ culpanda, vicissim

Illa, prius, cretâ; mox hæc, carbone, *notamus*.—PERSIUS.

### DIVISION I.

### ENGLISH.

ART. I.—*Medico-Chirurgical Transactions.* Vol. XIII. Part I.—8vo. pp. 280; with five Plates. Longman and Co. London, 1825.

AFTER a long and rather an alarming interval, we find another volume of these Transactions once more upon our table,—an event which we conjecture to be mainly owing to the activity of the new secretaries. Much, no doubt, depends upon these gentlemen, and it would have been impossible for them to have accepted of office at a period when zealous exertion was more required: indeed, immediate dissolution seemed impending; and it is even yet questionable how far the Society can be rescued from that state of lethargy into which it has for some time been gradually declining. A temporary stimulus, however, has certainly been given, and we must hope that an entire resuscitation may yet be effected.

The volume, as on former occasions, contains a considerable number of cases, and comparatively few essays; and these, if not such as to give any additional reputation to the Society, are at least such as will not lessen the respectable character which their Transactions have hitherto borne.

1. *Case in which the Subclavian Artery was successfully tied.* By Mr. KEY.

This was a case of axillary aneurism, occurring in a man aged thirty-six, an extra tide-waiter at the Custom-house, who, while making a sudden exertion with his right arm, felt something snap below the collar-bone. In a few days a small tumor was perceived on the fore-part of the chest, about an inch and a half below the clavicle: it pulsated strongly, and gradually increased. This occurrence took place in July, and on the 29th of August he applied at Guy's Hospital as an out-patient. Soon after this time the aneurism increased considerably, extending down the axilla, with unceasing pain and great constitutional derangement. This unfavourable change was produced by attempting to reduce the tumor by pressure, which had been done in the intervals of his seeing Mr. Key. The operation being resolved upon, was thus performed:—

"The patient being brought into the operating theatre, the extent of the sac in the neighbourhood of the subclavian artery was ascertained, to guard, if possible, against the danger of opening it during the operation of passing the ligature under the vessel: it appeared to be bounded above by the subclavian muscle, and the artery above the clavicle appeared (as far as the finger could ascertain) to be healthy. The clavicle was raised to its utmost, and was curved considerably backwards towards the trapezius muscle; it could not be raised higher by pressure made upwards against the elbow. The patient being laid upon an inclined plane, so that the light from a large skylight might be thrown into the triangular space in which the artery lies imbedded, I began the external incision in the following manner:—Standing by the patient's right side, I drew the integuments down over the clavicle with my left hand, and cut freely upon the bone, beginning about half an inch over the clavicular portion of the sterno-mastoid, and continuing the incision outwards for three inches. The integuments being relaxed, the incision became raised above the third of an inch above the clavicle, and exposed a strong platysma myoides, which was divided to the same extent. Numerous turgid veins were now exposed lying upon the cervical fascia; to avoid them was impossible; they were therefore divided, and about three ounces of blood were quickly lost: one larger than the rest was secured by Mr. Travers, to prevent any obstruction from hemorrhage in the after-steps of the operation. The dense outer layer of the cervical fascia was then more freely divided, and the loose cellular texture enveloping the glands of the neck being detached by the finger, the omo-hyoideus was laid bare. A little further dissection with the end of a director exposed the artery to the finger, pulsating over the rib; but the depth of the angle in which it was inclosed rendering it impossible to pass a ligature under it, about three-quarters of an inch of the clavicular portion of the sterno-mastoid was divided, which afforded sufficient room, and rendered the concluding part of the operation easy. The artery became readily exposed to view, and an armed aneurismal needle was passed with facility under it. A single ligature of silk was tightened around the vessel, and the edges of the wound brought into contact with two sutures and adhesive plaster. The patient had been so little exhausted by the operation, which lasted twenty minutes, that he expressed a wish to walk down stairs to his bed; which, of course, was not consented to.

"The absence of any very untoward symptoms, renders it unnecessary to trouble the Society with a detail of the conclusion of the case. It will be sufficient to mention that, eight and forty hours after the operation, a general excitement of the system, with an acceleration of pulse, induced Mr. Travers to prescribe (during my absence from town) a purgative of scammony and calomel, which not only removed the symptoms of general fever, but also a retention of urine, arising from a slight stricture, under which he had previously laboured. His urine had been twice drawn off by the introduction of the catheter. A trivial irritation of the trachea, inducing an occasional cough with expectoration, was allayed by the opiate linctus of the hospital. Opiates in other forms were not found necessary, with the exceptions of two nights on which his rest had been broken, when gtt. xxv. tinct. opii were given him. The œdema of the limb subsided quickly after the ligature was applied to the vessel: its natural warmth was maintained, perhaps aided by being enveloped in a double fold of thin flannel; and the pain, of which he complained prior to the operation, altogether left him as soon as he returned to his bed. The local treatment consisted in the application of a light poultice to the wound, after the removal, on the fourth day, of the adhesive straps and sutures; and in the prevention of sinuses from the lodgment of purulent matter.

"The ligature was found lying detached in the wound, on Wednesday morning, October 1st, the twelfth day after its application, and was removed without hemorrhage. The wound was nearly cicatrised, with the exception of the aperture occasioned by the ligature, which is gradually closing. On the following day he was allowed to leave his bed, and walk about the ward, to relieve the fatigue occasioned by the uniform position he had scrupulously maintained, till the ligature came away. The tumor is gradually subsiding, gives him no uneasiness, and promises to be absorbed without inflammation or suppuration. The pulse at the wrist cannot yet be felt." (P. 4—7.)

Mr. Key points out two circumstances, which he regards as greatly facilitating the operation: one is the division of the clavicular portion of the sterno-mastoid muscle, and the other is the mode of conducting the needle under the artery, and raising the end of the ligature; but, as the thorough understanding of this requires the assistance of a plate, we must refer to the paper itself. It is added, in a postscript, that the patient has nearly regained the use of the arm; the tumor has disappeared, with the exception of a little hardness. The pulse at the wrist has not returned.

2. *Case of Tumor in the Anterior Mediastinum, containing Bone and Teeth.* By Dr. J. A. Gordon.

A very stout young woman was admitted as a patient at the Islington Dispensary, in June 1822, labouring under symptoms of pneumonia. These were subdued by the usual means; but a harassing cough and frequent pulse remained, and resisted every remedy. In August she pointed out a small round tumor below the sternal extremity of the left clavicle: it was the size of a nut, and pulsated strongly. This was regarded as an aneurism, and the patient treated by free blood-letting and spare diet. In the course of three weeks the tumor rapidly increased, and rose above the clavicle; after which it again somewhat subsided.

On the 7th of November she was removed to St. Bartholomew's Hospital; from which she was again dismissed in December, for irregularity, and returned to the Dispensary. The tumor remained stationary for some time, but, in the spring of 1823, it gradually extended over the trachea: in the middle of June it began to point, and burst at the right side of the sternum on the 23d, discharging only serous fluid. On examination with the probe, this was found to be a small superficial sac, and the opinion that an aneurism existed underneath was still retained. No discharge took place after the 1st of August, when the tumor began to recede, and by the 12th of September no trace of it remained. The patient now went to work in better health than she had for two years.

On the 17th of October she again came to the Dispensary, with symptoms of general fever and oppressed breathing, but without local complaint. On the 20th she died; and the following notes of the post-mortem examination were made by Mr. Kingdon, one of the surgeons to the institution.

"There was a tumor in the anterior mediastinum, closely attached to the upper two-thirds of the sternum and the sternal extremity of the right clavicle. The left side of the chest contained a considerable quantity of fluid, and the lung was adherent to all such part of the costal pleura not so occupied. The right lung was adherent on its whole surface, and not capable of being detached at any part: its interior was loaded with fluid, and offered the appearance of œdematous cellular tissue, resembling lung only in its colour. The heart was flaccid, but its interior arrangement apparently healthy. The aorta and vessels given off at its arch were healthy; but the arteria innominata was completely enveloped by the thickened cellular tissue which connected the tumor with the surrounding parts. The parietes of the tumor participated in the character of that with which it was in immediate connexion: thus its anterior, from which the sternum was with difficulty raised, had the close compactness of tendinous expansion; and its posterior and lateral portions were more loose and flaccid. The contents were serous fluid, sebaceous matter, mixed with hair, (the latter not in large quantity, nor in distinct locks,) and an apparently fatty mass at the bottom, which being cut open proved bony; and, on more careful examination, a bone was detected very nearly resembling the upper maxillary, a portion of alveolar process, which might seem to belong to the upper or lower maxillary, and seven teeth (two cuspidati, two incisores, and three molares). One of the cuspidati has its crown perfectly covered with enamel, and freed from its capsule; the other is covered with the capsule, but is removed from its socket without any connexion. The molares are in their sockets, imperfectly formed; while the incisores are, by means of their capsules, attached to what at first appeared fat, but which, on closer examination, seems to possess the character of palatine membrane." (P. 14—16.) [illegible]

3. *Case of Injury of the Blood-vessels, producing pale dry Gangrene of the Foot.* By Mr. T. W. Chevalier.

The injury is thus described :—

"On Friday night, September 5th, 1823, Mr. T. M—, aged thirty-two years, received a blow in his left groin, from the shaft of a van, against which he rode with great violence. At the time of the accident, he is stated to have lost about a pound and a half of dark-coloured blood, and to have been taken up senseless. In about half an hour I found him sensible, but exceedingly pale and restless, and complaining of great pain in his leg and foot. His pulse 120, and feeble. The external wound was about three inches long, and parallel to Poupart's ligament. On removing the coagulated blood, the femoral artery and vein were exposed for upwards of an inch of their course; the former vessel pulsating forcibly, the latter fully distended. There was now only an oozing of venous blood. My finger passed readily into a cavity extending for more than two inches directly backwards, on the inner side of the great blood-vessels, and on a level with the origin of the profunda ; for about the same extent towards the anterior superior spinous process of the ilium ; and for three inches between the integuments and the fascia on the inside of the thigh. I considered it impossible to heal the wound by the first intention ; it was loosely lined with lint, and its edges slightly approximated." (P. 17, 18.)

Next day he had lost all feeling in the lower half of the leg, which was "cold, pale, and cadaverous." No pulsation could be felt in the arteries of the limb ; and, on the 7th, the coldness had extended as high as the ligamentum patellæ, and a slight discoloration had begun to appear round the limb at this part. Up to the 14th, the discoloration occupied nearly the same extent, having spread a little on the inside, and having become throughout of a deeper hue. The warmth of the hand, applied to the metatarsal joints or heel, was still perceived : there was no vesication, nor any sign of approaching inflammation, at the edge of the discoloured part, except great tenderness. The colour of the calf of the leg was like that of a bruised part, and did not return when dispersed by firm pressure; so that, on the morning of the 14th, Mr. Chevalier discovered the pale spots which he had made by pressing with his finger the night before. The ankle and foot were like new mahogany, and this colour could not be removed by any degree of pressure; the toes were dry and shrunk, but did not lose their cuticle. From the 15th the patient lost ground, having an attack of fever every afternoon ; during the hot stage of which he generally lost an ounce or two of blood from the wound, as well as occasionally at other times, on making any exertion. The wound itself began to assume an unhealthy appearance, and the sensibility of the limb diminished. On the 20th the wound looked better, and continued to do so till the 23d, when a coagulum of blood, as large as a walnut, and beginning to have its surface broken by putrefaction, was pressed from the bottom of the wound. An alarming hemorrhage followed, and the vessels were with great difficulty secured. The patient died at midnight.

"*Dissection.*—The arteries were all impervious in the neighbourhood of the wound; but the inguinal, the femoral, and the profunda veins were found separated by ulceration, and terminating by open mouths in the depth of the wound. These vessels had all been tied, so that the patient died from constitutional debility, the effect of an intemperate life, of the injury in his groin, and of the loss of blood previous to the operation, with that of about a pint and a half which escaped rapidly while it was being performed. The muscles of the leg were rather livid when first exposed, but not at all disorganised. It is remarkable that, although there were some traces of florid blood in the anterior tibial artery, the femoral was found distended, below the seat of the wound, with blood of the deepest modena hue. The colour of the calf of the leg was become very much darker since yesterday morning.—(P. 23, 24.)

### 4. *Case of Ulceration and Rupture of the Stomach.* By Dr. Elliotson.

On the 1st of March, 1824, Dr. Elliotson was called to a lady, about forty years of age, tall, thin, and of the melancholic temperament. Her situation is thus forcibly described:—

"She was standing in her bed-room, leaning on the neck of a female servant, and groaning with agony at the pit of the stomach. Her features, naturally long and sharp, seemed now unusually so; her swarthy pallid complexion was become cadaverous, and the expression of her countenance was that of the most dreadful suffering. She was shivering with cold, though before the fire, and her hands were icy and blue. Her pulse was 120, but neither full nor hard, nor particularly weak. Her breathing was very quick and short. She retched every now and then, but vomited nothing more than the barley-water she was drinking." (P. 26, 27.)

She had been in this state rather more than an hour. She first complained of chilliness for a few minutes; and, having fixed her eyes on vacancy for a minute or two, suddenly cried out with pain at the pit of the stomach. She had been exposed to cold the preceding day,—had dined on pork (which she asserted agreed with her better than any other kind of meat,) a few hours before,—her bowels had been open in the morning,—she had no hernia. Sixty drops of laudanum were administered; and, being rejected, the dose was repeated three times at intervals, but without affording relief. There had been tenderness to pressure in the epigastrium from the first; and, as this evidently increased, Dr. Elliotson bled her to the extent of about twenty ounces. This produced no faintness, and gave no relief; neither did the blood become either cupped or buffy. Sixty drops of laudanum were now again administered; soon after which she was considerably better. Next day she continued better; but, as there was still much tenderness of the abdomen, a large blister was applied, and ten grains of calomel ordered.

On the third day the tenderness was aggravated, and extended all over the belly; the pulse still 120, but small and feeble; the coldness, anxiety of countenance, &c. rather aggravated; the tongue clean; bowels not opened; she had great thirst, and had been constantly drinking "water so hot, that no one but herself could hold the glass." Five grains of calomel were prescribed, to be taken every three hours till the bowels were evacuated; and enemata, each containing three ounces of sulphate of magnesia and six drops of croton oil. In the evening, no benefit having accrued from these means, a drop of the croton-oil was ordered every four hours, and an injection containing twelve drops. Next morning it was found that three pills had been taken and retained, and that the enema had been given: the bowels had acted once. Her constant pain was "infinitely less;" she bore pressure better; but her pulse was 140, and she was restless. She had several stools during the day; and died at five o'clock, perfectly sensible.

The following appearances presented themselves on dissection:

"The abdomen was prodigiously distended, and, on opening the peritoneum, a large quantity of very fetid gas escaped. On the whole parietal portion of the peritoneum was a layer of fibrine; as also on the whole convex surface of the liver, the anterior surface of the stomach and of the omentum, and on much of the intestines. It was for the most part readily peeled off, but had effected adhesions between some portions of the small intestines, and between some portions of the peritoneum and omentum. A good deal of yellowish fluid, with white flakes, was collected in the upper part of the abdomen, such as is the mere product of inflammation; but I could discover no effusion of the contents of the stomach. In the anterior part of the cardiac half of the stomach, a little below the small curvature, was a perfectly circular aperture, with a smooth edge, large enough to admit the end of the little finger; and the surrounding part was of a dark colour, to some extent. The stomach contained a good deal of soft dark matter, which readily escaped on moving or pressing the organ. On examining the interior, a large ulcer was discovered, two inches in length, broad at one extremity and gradually narrower towards the other, with smooth edges; and the surrounding parts were greatly thickened, and very red and hard. The ulceration was gradually deeper from the narrow extremity to the other, where the rupture had occurred." (P. 30, 31.)

The diagnosis given by Dr. Elliotson is of importance, as contrasted with that laid down by Mr. Travers.

"From the dreadful agony which was felt from the first,—its sudden commencement, and that at the pit of the stomach, and its greater intensity there throughout the disease than in any other part of the abdomen,—the ghastliness of the countenance, and her icy coldness, even when I first saw her,—the absence of fulness and all hardness in the pulse, even during the evident existence of intense abdominal inflammation,—and the rise of this at the epigastrium, and its diffusion all over the abdomen,—I apprehended the stomach was ruptured, and I very earnestly requested permission to examine the body.

"In an interesting paper on cases of this kind, published in the eighth volume of our Transactions, Mr. Travers has stated the chief diagnostic symptoms to be—1st. Sudden, most acute, peculiar, and unremitting pain, radiating from the pit of the stomach, or navel, to the circumference of the trunk, and even to the limbs; 2dly. Coeval rigidity of the abdomen; and 3dly. A natural pulse for some hours, till the symptoms of peritonitis begin.

"In this case the pain did remit: after the fifth dose of opium, she became comparatively easy, and remained so for twenty-four hours. The rigidity of the abdomen did not strike me. The pulse was 120 from the first." (P. 29—32.)

Dr. Elliotson refers to the cases published by Dr. Abercrombie, in the Edinburgh Medical and Surgical Journal for January 1824; and to a case by M. Laennec, in the Revue Medicale for March of the same year. In addition to these, we beg to mention that an interesting case is published by Mr. Griffiths, in the fifty-third volume of this Journal.

5. *A new Variety of Extra-uterine Pregnancy.* By M. Breschet.

We are glad to find the name of this distinguished pathologist among the contributors to our Transactions, and hope to see the example followed by others of his countrymen. His paper details at some length a new form of extra-uterine development of a fœtus. We must content ourselves with merely mentioning its nature, and giving the conclusions deduced by M. Breschet. We do this the rather as the peculiarity of the case cannot be detected before death, and as the occurrence is one extremely rare.

There are three varieties of extra-uterine conception, the existence of which must be known to our readers:—1st. Where the embryo has passed into the cavity of the abdomen. 2d. Where it is developed in the ovarium. 3d. Where the development takes place in the tube; the ovum having left the ovary, but not having reached the uterus. Now to these M. Breschet has added a fourth variety, in which the fœtus is inclosed in the parenchema of the uterus itself, except that it is separated by a cyst from the substance of the viscus, no communication existing between the cavity containing the fœtus and the cavities, either of the uterus or abdomen. Some cases of this kind are related, and the following conclusions drawn:—

"1st. That extra-uterine pregnancies may occur in the substance of the uterus itself.

"2d. That in this, as in all the other varieties of extra-uterine pregnancy, gestation arrives slowly and with difficulty at the ordinary period; and I think that, in this variety, the ovum must experience more difficulty in its development, than when it is in the fallopian tube.

"3dly. That, at a period difficult to determine, a rupture takes place, accompanied with effusion of blood into the abdomen, and death is the quick and inevitable consequence.

"4th. That the membrana decidua exists in the uterus before the arrival of the germ; since, in all my observations, this membrane was distinctly formed, although the tubes were obliterated.

"5th. That the membrana decidua does not belong to the embryo, properly so called; and that it is not indispensable to the nutrition of the fœtus.

"6th. That the uterus may be developed, and its cavity enlarged, without the presence of a fœtus in the usual place.

"7th. That the placenta is not always formed in the human species, so as to constitute a uniform mass or cake, but that it may be developed at different distinct points of the ovum in the form of vascular penicilli, as is seen in ruminating animals: the solipeda, for example. (P. 49.)

6. *Case of Fallopian-Tube Pregnancy.* By Dr. ELLIOTSON.

This case occurred in a robust woman, aged thirty, who had been married a year and a half, but believed she had never been pregnant. She had experienced nausea for a fortnight, when she was suddenly attacked with violent pain low down in the belly, and accompanied by vomiting. These symptoms intermitted for about three hours, and, then returning, continued till her death, which took place forty hours from the commencement of the attack. During the last day and night, there was extreme tenderness of the abdomen; the pulse was 120, and very weak; the countenance deadly pale; there was frequent syncope, and she was extremely restless.

A large quantity of blood, and an embryo about the size of a gooseberry, were found in her abdomen. The right fallopian tube seemed broken off near its fimbricated extremity.

7. *On the Ligaments of the Human Ossicula Auditus.* By Mr. T. W. CHEVALIER.

Of this paper we can only give the title, as constant reference is made to figures, without which it would be impossible to make the description intelligible. Indeed, the plate is not given, the drawing, we are informed, having been mislaid: it is promised in the second part of the volume, with some supplementary observations.

8. *Observations on the Saliva, during the Action of Mercury upon the System.* By Dr. Bostock.

Dr. Bostock, in some experiments which he performed many years ago on the saliva, detected in it two animal substances,—one very similar to coagulated albumen, and the other resembling the uncoagulable matter of the serosity of the blood. An opportunity having occurred of examining the saliva whilst the system was under the influence of violent mercurial action, he was desirous of ascertaining what change was thereby produced upon its chemical properties.

"The saliva was a light-brown colour, and had a faint odour; although slightly opake, it was nearly homogeneous; by standing for twenty-four hours, some small films and minute flakes subsided from it, by which its opacity was diminished: in this state it was made the subject of experiment. It was considerably adhesive, but only in a slight degree tenacious; it was easily transferred from one vessel to another in the form of drops, and, when added to water, immediately diffused itself through the fluid, and was completely incorporated with, and apparently dissolved by it. It did not indicate either acid or alkaline properties by the appropriate tests. By slow evaporation, until the residuum had acquired a degree of brittleness and a brownish-yellow colour, the quantity of solid contents appeared to be about one-fiftieth of the weight of the whole fluid.

"By being exposed for some time to the heat of boiling water, a degree of coagulation was produced; the fluid became considerably more opake, and thicker in its consistence, but there was no precipitate or separation of any of the solid matter. It was submitted to the following tests:—Solution of corrosive muriate of mercury produced a considerable precipitate, and, when the mixture was subjected to the heat of boiling water, a number of dense flakes separated from it, leaving the fluid transparent; after being passed through a filter, it had the aspect of pure water. By the addition of muriatic acid, the opacity of the saliva was considerably increased, and, by applying heat, a coagulum was formed, which gradually subsided; but it was less firm, and the separation was less complete than when corrosive muriate of mercury had been employed.

"From these experiments we learn that the chemical constitution of the saliva was considerably different from its natural state, and that this difference consisted in its containing a quantity of animal matter, possessing properties similar to those of albumen in its uncoagulated state, or as it exists in the serum of the blood.

"Having ascertained this change in the nature of the animal matter in this saliva, which it may be presumed was owing to the action of mercury upon the system, it became an interesting object of inquiry to ascertain whether any mercury could be detected in it. The method which I employed for this purpose was to treat the evaporated residuum with nitric acid, by which means any mercury, if present, would be converted into the nitrate, and to test the fluid by the proto-muriate of tin. A preliminary experiment was made, in order to learn how small a proportion of mercury might be detected by this process. A given quantity of mercury was converted into the nitrate: I took as much of this nitrate as contained one grain of mercury; this was successively diffused through different proportions of water, until it at length composed no more than $\frac{1}{10000}$ of the mixture, when I found that the protomuriate of tin produced a grey cloud, which was very distinctly visible. The same process was adopted with respect to the evaporated residuum of the saliva, but it did not afford the least indication of the presence of mercury. The experiment was repeated more than once without effect; and I may remark, that I obtained the same results in some experiments of a similar kind, which were performed several years ago.

"After an interval of sixteen days, I procured from the same individual a second portion of saliva: the use of mercury had, in the mean time, been omitted; and, although the quantity of fluid discharged was still considerable, it was much less than before. The sensible qualities of the fluid were now entirely changed: it was considerably opake, and had a number of mucilaginous flakes floating in it, which were insoluble in water, and not easily miscible with it. It was so viscid as to be capable of being drawn into threads; while, on the contrary, it did not admit of being dropped. Its solid contents, as ascertained by careful evaporation, were found to be considerably more in quantity than in the former case: it slightly reddened litmus paper, indicating the presence of an uncombined acid.

"When this saliva was submitted to the temperature of boiling water, it was rendered more opake, but no proper coagulation took place; after standing for forty-eight hours, there was a separation of a more dense substance from the remainder of the fluid, but in an imperfect degree only. The corrosive muriate of mercury and muriatic acid were respectively added to portions of the saliva: in each case the fluid was rendered more opake, but no distinct coagulation was produced; and although the effect was increased by applying heat, still the separation was not complete. By the addition of the subacetate of lead, a very copious precipitate was thrown down, consisting partly of large flakes; while the fluid was left quite transparent, and, as it appeared, deprived of all the animal impregnation. This saliva was examined, as the former had been, for the purpose of discovering whether it contained any mercury; and it is scarcely necessary to state that the search was unsuccessful." (P. 74—78.)

The change, it will be perceived, which was induced in this instance by the action of mercury, consisted chiefly in the conversion of the animal matter from the state of a mucous to that of a serous, or rather of an albuminous fluid. Dr. Bostock conjectures that, as those fluids which proceed from serous membranes appear to differ from the serum of the blood solely in the proportion of the albumen they contain, they may probably be generated by a process resembling transudation. In the secretions, however, of mucous membranes, a chemical change is effected; a new substance making its appearance, which did not previously exist in the blood. Now it would appear, from the case related by Dr. Bostock, that the operation of mercury upon these parts is to counteract the ordinary secreting process, and to reduce the action of the glands to that of mere transudation.

In two cases in which this distinguished chemist examined the fluid discharged from the stomach under acute inflammation, the secretion from the mucous membrane appeared to be much more albuminous or serous than in its natural state. One was supposed to have been poisoned by arsenic; and in the other, the individual died a few hours after swallowing a large quantity of ardent spirits. Another coincidence alluded to by the author, is the fact that certain morbid states of the urine, in which it is found to contain albumen, are generally regarded as indicative of inflammatory action.

In an Appendix is added an account of some experiments on the mucus expectorated by a patient suffering from catarrh. In this case the saliva had undergone no essential change in its chemical composition; but the quantity of the substance which may be regarded as its specific ingredient was much increased in quantity. There was no tendency to that change in its nature which is produced by the action of mercury.

9. *Case of Fungus Hæmatodes of the Brain.* By Mr. J. HUNTER.

This case is minutely and well detailed, but does not appear to us of sufficient importance for quotation.

10. *On a new Preparation of Croton Tiglium.* By the late Mr. POPE.

Mr. Pope states that the medulla is the part universally employed in India, and that it is administered in substance as the usual purgative of the natives, who carefully separate the husk and epidermis, and also take out the eye of the seed. The medulla thus prepared is then mixed with a preparation of antimony, and formed into pills. This mode of employing the seed agreeing with the results of the author's observations, he was induced to subject them to a similar preparation, and has by that means procured an oil, which is submitted "with some confidence to the medical world." The best mode, however, of exhibiting this oil is in alcoholic tincture; and the following method of preparing it is recommended:—

"Take of the seeds of croton tiglium, carefully deprived of the husk and epidermis, and bruised, two ounces; alcohol (sp. grav. 836°) twelve ounces. Digest for six days, and strain. The dose of the filtered tincture for an adult is about twenty minims.

"The oil of tiglium is soluble in ether, and in oil of turpentine. It is only partially soluble in alcohol, which dissolves rather more than two-thirds of it, but takes up the whole of the purgative principle; the residuum exerting little or no action in the intestinal canal." (P. 100.)

[To be concluded in our next Number.]

---

ART. II.—*A Treatise on the Properties and Medical Application of the Vapour Bath, in its different Varieties, and their Effects in various Species of diseased Action.* By J. GIBNEY, M.D. of the University of Edinburgh; Resident Physician at Brighton; and senior Physician to the Sussex County Hospital, and General Sea-Bathing Infirmary.—8vo. pp. 156. Knight and Lacey, London, 1825.

IN our former Numbers, we have frequently had occasion to advert, and direct the attention of our readers, to the medicinal effects of vapour and gaseous baths; and, as their utility is of growing importance, we took up Dr. GIBNEY's volume in the expectation of eliciting practical and useful deductions from its perusal, not being strangers either to his talents or professional attainments. This expectation has not been disappointed; though we may, perhaps, be allowed to say, that we do not give this opinion without some qualification, the reasons for which will become apparent as we proceed in our analysis of the work.

The first four chapters are rather of a popular nature: they contain a pleasing and general review of the luxurious modes of bathing, percussion, and shampooing, which are practised in the warm climates of Turkey, Egypt, Persia, and other countries where the learned sciences have not yet penetrated; as well as the modes of bathing in practice among more enlightened nations, as France, Italy, Germany, &c. This portion of our author's labours evinces a considerable share of reading, and proves that he has left few sources of information unexplored.

We are tempted to extract a passage from Dr. Gibney's fourth chapter, wherein he explains the effects of vapour upon the animal economy. After detailing some of Mr. LESLIE's experiments upon the properties of heat, he goes on to say—

"These facts are of considerable moment, as they clearly show the wide distinction between vapour and heated water, in their separate application under the form of a bath; and as in the one heat is conveyed from a dense medium, containing it in an under proportion, and from the other a highly rarified medium, possessing it in a superabundance, and imparting it with great facility, not only to the external surface of the body, but to the most minute air-cells of the lungs, at the same instant, the effect should be considered very different indeed, and is consequently much greater upon the vital functions and on diseased action, in the generality of cases; and, further, this view of the subject may serve to illustrate the general effects of the medium in which we exist, and the changes that may be induced by the application of vapour of various degrees of temperature and tenuity. Hence, when steam or vapour is diffused over the surface, and brought to exercise its full powers under proper regulation, the lungs expand with greater freedom,—a succession of favourable changes often ensues, arising from their immediate connexion, and dependent upon the invigorated condition of the skin, and the rarefied state of the medium then breathed.

"The diminished pressure of the surrounding vapour, added to the active agency of heat, which is imparted under this form with great freedom, act like cordials to the stomach, imparting vigour and health; while the process of circulation and absorption proceed with energy and facility, the animal spirits become at the moment more exhilarated, and a pleasing and luxurious sensation pervades the whole system.

"The unison and sympathetic consent between these two great exhaling surfaces is such, that a free and healthy expansion of the one is certain of producing a like and simultaneous condition of the other; and a regular exercise of their functions is so essential to a general salutary action, that, where they become irregular or deranged, a state of disease is inevitable; but, when performed agreeably to the ordination of animal life, those processes, on which depend the various secretions and other functional exercises of our wonderful existence, proceed with vigour.

"This organic sympathy, and the existing chain of connexion, is admirably and nicely balanced, so that, on the accession of disease in an internal organ of any importance, its presence is soon betrayed by a deranged action on the surface. On the contrary, where the vicissitudes of climate, or other causes, occasion an irregular action on the skin, the internal viscera, in one way or another, manifest disease in a greater or less degree." (P. 46—49.)

The details contained in this portion of our author's work are to be considered, however, as only prefatory to the succeeding chapters; and accordingly we find, at chapter the fifth, that the description of the various kinds of vapour and medicated baths is entered upon: and here we regret that he has not been more explicit on this part of his subject. He does not, we conceive, sufficiently point out for what diseases, or in what stages of disease, he advocates the employment of this remedy. The profession is not unacquainted *generally* with the curative efficacy of one or other of these baths, but they now look for some data which may regulate them for the future in the adoption of either. In some cases of internal inflammation, and inflammation of the larger joints, we have certainly known the vapour-bath do harm, where we have expected its effects to have been soothing and salutary; and are led to conclude, from the observations of our correspondent, Mr. Green, that the inflammatory action had not been sufficiently subdued by depletion; and that the heat occasioned by the vapour bath, under these circumstances, had produced too much excitement, and consequent aggravation of the symptoms. Here, then, is a practical deduction, which may assist us when directing this remedy.

The vapour baths used at Brighton would seem, from the papers that have at various times appeared in the Medical Journals, to admit of much improvement in their construction and application; and, although we are not disposed to controvert the good that may have been there effected, yet we think that good might admit of increase under different circumstances: and we say this in consequence of witnessing the benefits received at Mr. Green's establishment, the principle of which bears little resemblance to any thing of the kind in Brighton.

With respect to the medicated and aromatic vapour baths, we suspect there is but little medical virtue in them, and that they are not superior to the mere vapour of boiling water. The fumigating baths require to be considered in a very different light; they are either powerfully efficacious, or proportionally mischievous. The hot air bath seems to have many advantages over the vapour bath, to which we would direct the attention of Dr. Gibney. We are somewhat surprised, also, that in mentioning the sulphureous bathing, as practised by Dr. Gales, of Paris, in the hospital of St. Louis, he should have spoken of them as lately introduced into this country, and practised upon an improved plan by Dr. Dick. Who is this doctor? and where is this establishment?

In the same chapter, our author mentions the partial application of vapour, as employed by M. Rapon, of Lyons, and which he calls a *douche de vapeur*, "in the cutaneous complaint commonly called ring-worm, ophthalmia, some diseases of the ear, and many other local affections, with great advantage: by this means, any part may be instantly blistered with the greatest facility, which in some urgent cases is an object of important consideration." We conceive that the partial application of vapour may sometimes be beneficial, but are advocates for its more general application in preference, since complaints strictly local are seldom met with; and this has been long stoutly contended for by a medical luminary of the present day.

Mercurial fumigation is next considered, and it is to be regretted that its employment has not made much progress in this country: this may have arisen from the difficulty formerly attending their administration, but now that these baths are so much improved in construction, we should hope that they would come into more general use.

Dr. Gibney informs us that—

"The electrico-aeherial baths, used formerly by Mr. Lawnds, and now, upon an improved and very efficacious plan, by Mr. Adams, 22, Ludgate-street, are deserving of particular attention, as the powers of the electrical apparatus are such as to command the full influence of the electrical fluid, under all its forms and modifications, without the usual difficulty and labour in its production. By this ingenious and judicious contrivance, there is no effect to be derived from electricity that is not under command, and rendered applicable to every medical purpose that may be required, with the greatest facility." (P. 71, 72.)

Our author next speaks of a mode of promoting absorption and suppuration, by means of hollow tin cases filled with hot water and lined with felt, and so adapted as to be applicable to the chest, body, or limbs. The heat is kept up by renewing the hot water. For the promotion of the above-named objects, Dr. Gibney says this method is preferable to the usual means now practised, and the application of cataplasms.

In his sixth chapter, our author mentions, with due praise, the sudatorium of Dr. Gower: this has been long before the profession, and we do not think it necessary to do more than to say that it should not be taken in the recumbent position; for, when the patient becomes under the influence of the bath, too great an accession of blood is occasioned in the head and chest; and this remark equally applies to any form of the vapour bath.

This chapter concludes with a description of shampooing, or massing, as it has been called. It is used in Egypt, as well as in India, as a sort of salutary process, by persons in health, as well as in diseases of the joints or elsewhere; but it can only be serviceable in cases of rigidity or stiffness, or where there is an absence of inflammation.

Chapter the seventh is devoted to the consideration of the advantages of friction and percussion; and here our author quotes largely from Dr. Gower's tracts, who has not forgotten to mention the authority of Celsus, nor the reputation of Asclepiades, who claimed the merit of the first invention of friction as a remedy in disease. This leads to a description of an instrument, called by Dr. Gower the pulsator, which (as our readers are probably acquainted with it,) we shall pass over.

We now come to the application of the vapour bath; and, after some preliminary remarks, we are told—

"Its action kindly solicits the fluids to the surface, and at once frees the circulation and sooths the sensations, so that relief may be expected where a disordered condition of either has for any time existed; for the vital organs being but too frequently in a morbid state, from unhealthy cutaneous action, it may be truly said that the influence of cold in producing disease is not greater than that of heat, under the form of vapour, in mitigating and removing its baneful consequences.

"By its proper application, it will be found useful and efficacious in those cutaneous complaints so generally known, and which add to the intensity of the other affections with which they chance to be conjoined, and, from so extensive an outlet as that of the skin, are soon mitigated; also in many of our most formidable chronic diseases, such as gout, in most of its varieties, rheumatism, paralytic affections, hydropic complaints, diabetes, female obstructions, scrofula and glandular diseases, dysenteria, congestions and obstructions of the liver and spleen, occasioning their torpor, and the diseases that follow in a connected chain with the stomach and alimentary canal; on the condition of which the healthy or unhealthy state of the whole animal economy has so direct and considerable a dependance, and on which the primary effects of all internal remedies must be exercised, before their influence can be diffused over the general system.

"To the above enumeration, we should not omit febrile diseases, under the modifications of typhus and intermittents, and certain conditions of scarlet fever, where vital reaction is oppressed with disease and considerable debility. Here, from the difficulty of moving the patient, vapour from the spirit-lamp will prove most suitable, as has been described in the foregoing part of this treatise." (P. 97—99.)

The eighth chapter commences with an enumeration of the particular diseases in which the vapour bath is applicable; and we have been impressed, upon reading this account, with the great number and importance of these, including atonic gout, rheumatism, epilepsy, nephritic affections, &c. Dr. Gibney here gives a very necessary and forcible caution as to the rash and indiscriminate use of this remedy. Great care, he observes, should be taken to ascertain whether general or local bleeding, together with purgatives and deobstruents, should not precede the active use of the vapour bath; for, on this subject, experience has, in many instances, instructed us that much mischief and considerable danger has arisen from a want of the necessary precautions, and a remedy, in itself of very great value and importance, has but too often suffered in its character, by invalids entering upon its use without due consideration and circumspection.

Some useful practical rules follow this judicious advice. The bath is best taken in the morning; and it is advisable, during its employment, that the diet should be light and nourishing, and that early hours should be carefully attended to.

"During the winter months, patients should be cautious in being cold or chilly immediately before entering into the bath; to obviate which, brisk exercise, for some time previously, will serve the best purpose. On the contrary, in the warm season, their blood, from one cause or another, should not be over-heated. Extremes, under such circumstances, but too frequently counteract the advantageous consequences that might otherwise ensue. It is proper to observe, that it is much more prudent and wise to use the necessary precautions against cold after an exposure to vapour, than to immediately hazard any risk. This caution applies more to women and children than men, whose constitutions differ so very materially from those more delicately formed. The vapour bath, when favourably applied, occasions the cutaneous glands to throw forth, on the surface, whatever foul matter may obstruct the free exit of perspiration; while, by subsequent ablution with soap and warm water, instantly applied, the sordes are most effectually removed, leaving the skin smooth, and its transpiration unimpeded. A sense of comfort and vigour is thus imparted, and the vital and animal functions performed with strength and energy." (P. 105-6.)

The remarks upon the mode of employing the vapour bath in particular diseases occupies the remainder of this chapter and the whole of the ninth. We shall make an extract or two from this portion of our author's labours, premising that the space we have to devote to this analysis does not permit us to do much more than recommend these observations to our readers. Speaking of the effect of vapour bathing in gout, Dr. Gibney says—

"Using the vapour bath in gouty cases, when the stomach is most empty, often succeeds, when a different practice proves unavailing. When taken before breakfast, this frequently happens; and, from the history of the disease, according to the best received opinions of its character and symptoms, this might naturally have been expected, was it not that the remains of a dangerous and false theory, founded upon the humoral pathology, still exists in the minds of both patients and practitioners." (P. 111.)

Respecting its application to scrofulous cases, we find the following passage:—

"In glandular swellings, whether in a state of ulceration or not, the good effects succeeding to the general and topical application of the vapour bath are often very remarkable, after the failure of a regular course of cold and warm bathing. This happens but too frequently in those obstinate strumous tumifications of the knee and other larger joints, called *white swellings:* in such cases, however, the state of the bowels and digestion will generally be found irregular; a matter of great consequence to be attended to, while any hopes of relief are entertained from the usual means, in conjunction with the bath." (P. 129.)

The portion of the work devoted to cutaneous diseases appears to us rather too limited: we think Dr. Gibney might extend this part of his work with advantage, by pointing out, in rather a more particular manner, those forms of skin-disease most usually and readily benefited by vapour bathing.

In the tenth chapter, our author pursues the subject of particular applications of his remedy, and advocates its employment in suspended animation, insanity, mercurial disease, and intermittent and scarlet fever. In the mercurial disease, we have known the sulphureous fumigating bath of essential service; and, in fevers of the most dangerous type, Dr. Gibney is of opinion that the vapour bath possesses powers over the febrile action, and, by promoting the free discharge of perspirable matter from the surface, is of the greatest consequence, even in fevers of the worst character, and particularly in those incident to warm climates, as it has the effect of calming and assuaging the most urgent symptoms. We imagine this remedy is but little known in our colonies; and as baths are now constructed, not only on an improved but a portable plan, we think our medical officers would do well to give this subject their consideration.

In conclusion, Dr. Gibney says, few diseases affect the human frame without the important functions of the skin becoming more or less concerned; and argues that this single fact should, in a degree, account for its almost general applicability, but more particularly in those diseases he has enumerated. A proper knowledge of its use, under any of those circumstances, will lead to its more diversified application in cases not particularly specified; but, as no active remedy can be said to be at all times exempt from disagreeable consequences, so it is with this; and, in order to ensure a successful issue to it, one general rule should never be lost sight of: the state of the head and the bowels should be well ascertained, and, as far as possible, all objectionable circumstances removed. Here we perfectly coincide with our author, and think with him that, in most chronic diseases, one or other of the baths of which he treats may be resorted to as an adjuvant, with every expectation of success. In acute diseases, we are of opinion that the profession will go on as they have been wont to do,—at least till they are more familiarised to the remedy, and it is more easily obtainable.

We conceive that the length and number of our extracts will sufficiently show that we have been pleased with Dr. Gibney's book, which we beg to recommend to the notice of our readers; and, as he appears to possess, together with many local advantages, the requisites for observation, we hope, at no distant period, to have again to discuss this subject.

### DIVISION II.

### FOREIGN.

Art. III.—*De la Membrane Muqueuse Gastro-intestinale, dans l'etat Sain et dans l'etat Inflammatoire; ou, Recherches d'Anatomie Pathologique sur les divers Aspects, sains et morbids, que peuvent presenter l'Estomac et les Intestins.* Par C. Billard, ex-Elève interne des Hopitaux d'Angers, &c.—Paris, 1825.

*Of the Mucous Membrane of the Stomach and Bowels, in the state of Health and Inflammation; or, Pathological Researches concerning the different healthy and diseased Appearances presented by the Stomach and Intestines.* By C. Billard, &c. &c.

This Essay, it seems, was crowned by the "Athenée de Medecine" of Paris, and we had ordered it for the purpose of analysis, when we met with a very full digest of its contents in one of the French Journals,* from which we have taken what follows.

The mucous membrane lining the alimentary canal is well known with regard to its structure, and certain varieties of texture in different parts of its surface. But descriptive anatomists have not been sufficiently clear with regard to the appearances of the membrane in its sound state; owing to which circumstance, we frequently find ourselves puzzled to distinguish between the healthy condition and one resulting from inflammation. This distinction, however, is very essential, and especially now when no post-mortem examination can be regarded as complete without an attentive and minute inspection of the intestinal canal. To arrive at this desirable point, the author first details a set of dissections, by means of which his object is to point out the various aspects of the mucous membrane in the state of health. He compares the consequences which result from this investigation, with what has been written by various authors on the subject; and, in conclusion, deduces the general conclusions resulting from this double source of information,—viz. reading and anatomical observation.

The mucous membrane of the stomach and bowels presents perceptible differences according to the age of the subject; and cases are related at some length to illustrate this. The information conveyed is interesting; but we shall take leave to confine ourselves to the point more immediately under discussion, and consequently to abridge the observations very considerably.

---

* *Archives Generales de Medecine*, Aout.

*Embryo, from four to five months old.*—The external surface of the tube whitish, and presenting very fine vascular ramifications; the mucous membrane of the stomach rose-coloured, and without furrows: this organ contained a small quantity of clear mucus. In the duodenum, the mucous membrane was of a reddish white, none of the valvulæ conniventes being yet manifest: the same appearance presented itself till the termination of the ilium, which had a brownish stain; this was capable of being removed by washing. Inner membrane of the cœcum a little rose-coloured; that of the ascending colon stained green by the meconium; the colour not removed by washing.

*Fœtus of seven months.*—Mucous membrane in the stomach thick, white, with a little rose-colour; in duodenum whitish, shaded with rose. Slightly marked transverse lines, indicating the site of the valvulæ conniventes. The jejunum and ilium of a rosy white; the inner surface of the large intestines greenish, from the meconium.

*Fœtus at the full period.*—Inner surface of the stomach presenting longitudinal striæ formed by the mucous membrane, which was of a light rose-colour; orifice of the pylorus white. The valvulæ conniventes in the duodenum composed of little rounded transverse lines, which disappeared on the slightest stretching of the tube. The mucous membrane throughout the whole of the small intestines white, shaded with rose; villous, but not so thick in proportion as in the two preceding. Two invaginations were found in the inferior portion of the small intestine, the mucous membrane being quite white in these parts. Large intestine filled with meconium, and internally green. The mucous membrane in this subject was easily detached from the surrounding parts. This fœtus was destroyed during delivery.

*Infant of twenty-two months.*—The subject of this observation was killed by an injury of the head, having previously enjoyed good health. The mucous membrane of the stomach not thick, but sufficiently villous; of a milk white, but smeared with clear mucus. At the cardiac orifice, the œsophagus was seen to terminate by a white festooned circle, which appeared folded upon itself. The valvulæ conniventes of the duodenum much developed; but there existed between the pylorus and first valvule a space as wide as two fingers' breadth, where the internal membrane was white and villous, but without furrows. The jejunum white, coloured with a yellow fluid, which, however, did not stain it. Same appearance in the ilium, at the end of which were numerous little elevations, white in colour, soft to the touch, and having the size and appearance of millet-seeds. The internal membrane, throughout the whole course of the tube, could only be detached in small portions, the torn edges of which did not bleed.

In this case it will be perceived there was no longer any of the rose-colour of the mucous membrane; the valvulæ conniventes were more distinctly marked, and the glands of the ileum more developed. The author proposes to designate by the name of *pylori-valvular* that portion of the duodenum which intervenes between the first valvule and the pylorus, and which is for the most part destitute of striæ.

*Girl of three years old.*—Died at the end of five days, of acute arachnitis. Mucous membrane of the stomach of a milk white, soft, and easily detached. In the pylori-valvular space was found a layer of mucus, beneath which the membrane was of a beautiful light rose-colour. The rest of the small intestines white.

*Child of eight years.*—This child was killed by a musket-shot. Inner membrane of the stomach of a milk white, thick, solid, and adhering strongly to the subjacent coats. The duodenum white, with a slight ash-colour. Much mucus in the small intestines, towards the end of which the ash-tint had disappeared.

A piece of artillery burst at Angers, during a public festival. Three children were killed, and the bodies examined at the hospital.

*The first, aged ten years*—was killed on the spot. Inner membrane of the stomach and small intestines white; the duodenum having a few yellow stains.

*The second, aged fourteen years*—killed by an extensive wound of the chest. Mucous membrane of the stomach white; that of the duodenum white, with a shade of ash. In the middle of the jejunum a yellowish tinge, for six fingers' breadth. In the ileum, the membrane whiter and finer than in the preceding portions. In the ileo-cœcal region, some injection of vessels; the cœcum of an ash-white.

*The third, aged sixteen years*—died some hours after receiving a wound in the region of the hip-joint: he had taken food just before the accident. The stomach filled with aliment; the inner membrane covered with very tenacious mucus, and of a general rose-colour, particularly at the great curvature. The duodenum was filled with a yellowish paste, the mucous membrane being of the same colour as that of the stomach. Beneath this membrane were perceived the blue trunks of veins, which did not give rise to any lateral ramifications. The jejunum was remarkable for the ash-coloured tint of its inner membrane, a hue entirely different from that of the stomach and duodenum.

*Young man, aged nineteen years.*—Killed by throwing himself from his window, and injuring the head; examined eight hours after death. Stomach filled with a quantity of food. The mucous membrane of the stomach of a rose-colour, with numerous folds, having different directions. Near the pylorus, a number of small glands, white, and soft to the touch: these parts covered with a layer of mucus. The pylorus white, suffering the fore-finger to pass. The membrane of the duodenum white, verging to ash; jejunum the same, having a yellow patch in the middle. In the ileo-cœcal region were found a number of glands, as at the pylorus. In the colon, the ash-tint described in the small intestines was no longer to be seen. Large portions of the mucous membrane could be removed at any part of the canal.

*Man aged twenty-eight years.*—Killed by an injury of the chest, which he survived an hour. Examination twenty hours after death. Stomach filled with food; the mucous membrane of a fine white, with a slight shade of rose; in the numerous furrows between which was abundance of mucus, having a shade of red. The duodenum white, and slightly ash-coloured. Towards the end of the ileum, three or four patches of bright yellow appeared in the midst of the white surface of the mucous membrane, and which resisted washing.

*Man aged forty-five years.*—Killed by a fall, which produced rupture of the aorta; examined fifteen hours after. Stomach containing food; mucous membrane white, shaded with red, having numerous striæ and abundance of viscid mucus. At the pylorus, the membrane whiter, and very villous; the duodenum having throughout an uniform light rose-colour, which was lost in the jejunum and ileum. The membrane of the great intestine white.

In the above cases, no reasonable doubt can be entertained with regard to the healthy state of the digestive organs, as the patients died of diseases unconnected with them. Three other cases are detailed, but these we omit, as the individuals laboured under complaints of some duration.

The general summary from the preceding observations is—first, that the mucous membrane of the stomach and bowels presents general characters, which are peculiar to it, and distinguish it from other membranes of the body; secondly, that it has characters peculiar to different ages; thirdly, that the colour varies according as the examination is made during or after digestion.

I. *General characters.*—The general characters regard principally the colour of the membrane, the folds or striæ which it forms, and the villosities and mucous follicles proper to its structure.

The colour of the stomach in the adult is usually whitish; that of the duodenum and jejunum white, verging to ash-colour: this ash-colour terminates at the conclusion of the ilium, and the membrane becomes whiter in the great intestine. The mucous striæ or furrows of the stomach have an irregular disposition, and do not always exist within the organ: generally they are most numerous at the great curvature, and co-exist with a contracted state of the stomach. As observed above, there constantly exists between the pylorus and the first of the valvulæ conniventes in the duodenum, a space as large as two fingers' breadth, where the membrane is destitute of fold or valvule. In the natural condition, the valvulæ of the duodenum, all lying in the same manner, and partly covered by each other, considerably resemble a surface covered with plates which overlap. Towards the end of the small intestine, these valvulæ become further separated, and more and more effaced.

The villosities are abundant in the stomach, particularly towards the pylorus: in this region they are usually grouped, a little flattened, and separated by very fine lines, similar to those on the skin of the hands. These villosities are less perceptible in the small intestines, and so little apparent in the large, that at first sight one would be tempted to doubt their existence.

The mucous glands may become developed in the sound state, and their mode of development, together with the variety of appearances which they give to the membrane, merits particular notice. It is difficult to compare the thickness of the internal tunic of the alimentary canal to any given measure,—the only way of judging of it is by the greater or less degree of its transparency: if, then, we tear off a portion, and lay it upon the finger, we see this as through a piece of white crape. In general, the membrane is thicker in the duodenum; less so in the stomach; then in the rectum, jejunum, ilium, and large intestine. In the stomach, it is thicker at the pylorus, at the cardia, and at the lesser curvature, than in other parts. We must take care not to confound the relative thickness of the mucous membrane with that of the entire parietes of intestine.

Certain other peculiarities of appearance in the mucous membrane might be enumerated, which are consistent with its healthy state, although not invariably found. Thus, the whitish circle which terminates the œsophagus encroaches more or less upon the stomach; accidental folds, crossing the direction of those which are natural, are occasionally found on the internal surface of the intestines; yellow patches, or bands of this colour, are observed; and worms induce no corresponding change of the membrane.

When the mucous follicles are developed, their mouths must be coloured for the naked eye to distinguish them; and the orifice, or tubercle, which corresponds to the opening of the ductus communis choledocus, or pancreatic duct, is not easily perceived, without pressing the duct so as to force out its contents.

M. Billard relates the case of a man, killed by a fall from a great height, in the stomach or duodenum of whom the folds and valvulæ conniventes were deposited in such a manner as to form square and lozenge-shaped spaces, as is observed in the second stomach of ruminating animals. Besides this, funnel-shaped depressions were easily seen with the naked eye in the cœcum, analogous to those described by Sir E. Home; but which he could not discern without the aid of the microscope.

II. *Varieties of the mucous membrane depending upon the age.*—In the embryo and fœtus, the membrane is rose-coloured, which is probably owing to the habitual congestion in the vessels of the abdomen; the inner surface of the great intestine is generally green. At this period of life, abundance of mucus lines the inner surface of the bowels. In infancy, the mucous membrane is of a milk white; of a less brilliant white during adolescence; and, in the adult, of a white verging to ash. This last shade is more striking in elderly persons, unless they are arrived at extreme old age, when the mucous membranes, in a manner, lose their colour, like other parts of the body under similar circumstances. If there be any impediment to the circulation, the venous blood flows back into the intestinal vessels, the blue-coloured branches of which are seen ramifying under the mucous membrane, a circumstance not uncommon in old persons.

III. *Differences during or after digestion.*—During digestion there is a sudden and transient flow of blood to the stomach, the presence of which gives a rose-colour to the mucous membrane. The duodenum, and occasionally even the commencement of the jejunum, participates in this colouring; but, except from this circumstance, it is never of a red tinge, as described by most writers. The other intestines afford no sensible varieties in their appearance.

It would appear that the blood thus accumulated in the mucous membrane during life, may remain there after death. Some of the cases quoted above tend to prove this; and M. Billard gives two remarkable facts in addition. A man hanged himself one evening in a public walk at Angers: next day the body was opened; the viscera were yet warm; the stomach, which was very small, contained three or four spoonsful of a whitish liquid, without smell; a white mucous substance formed a thin layer, which covered the furrows of the internal membrane, the colour of which was a light rose. Many lacteals were very apparent: on pressing them, the chyle, which was seen through their parietes, was made to flow out. An old gens-d'arme, affected with palsy of the tongue, and a slight mental derangement, died from suffocation, caused by eating too fast. On opening the body, which was done next morning, the mouth, pharynx, œsophagus, and even the larynx, were found filled with food; the stomach likewise was filled: the mass which this contained was softened and altered at the surface, but within, portions of beef and bread were recognised. The mucous membrane was of a fine rose-colour. These two facts are strongly in favour of the author's opinion respecting the state of the stomach during digestion.

*Opinions of authors relative to the aspect of the mucous membrane of the stomach and bowels, in its healthy condition.*—SABATIER has erroneously pointed out a reddish purple as the natural colour of the stomach. M. PORTAL justly remarks, that congestion of the veins may give a dark red to the lining membrane; but it is not correct to say that this colour is, in general, a mark of inflammation, as it may arise from causes entirely different. Neither is he correct in asserting that the jejunum is rather redder than the ileum. In the Anatomy of BICHAT, we find only very vague notice of the appearance of the inner membrane of the intestinal canal in the stomach and duodenum: it is said to be reddish, and it would follow, from other remarks, that it is thicker in the small intestines than in the stomach; an assertion contrary to what is advanced by M. Billard. GAVARD describes the inner surface of the stomach as grey, verging to red, but he says nothing of the colour of the intestines. BOYER, in calling the membrane of the stomach greyish, *(grisatre,)* seems to allude to that tint which the author before us describes as white verging to ash-colour, *(blanc legerement cendré;)* but Boyer adds, that this greyish colour has a shade of yellow or red. Now this last expression can only be correct with reference to the stain produced by the bile, and the redness produced by congestion; which, however, are but accidental circumstances. MM. CHAUSSIER, ADELON, and CLOQUET, assert that the stomach is a marbled red, and that the duodenum is reddish. M. Billard does not regard the marbled appearance sometimes presented by the stomach, as belonging to its healthy condition; but M. MARGOLIN thinks that the mucous membrane of this organ may have brown or blackish spots, without any other alteration of its texture. BECLARD says that the colour of these membranes varies from white to red. ALBERT MECKEL states that he never injected the villosities, without the mucus, and sometimes the whole contents of the canal, taking the colour of the injection; a phenomenon which may serve to explain the exudation of blood, which is occasionally found in passive congestions.

In concluding this part of the subject, M. Billard points out the resemblance and disagreement between the results of his observations, and those published in a posthumous Essay by M. Rousseau, inserted in the Archives Generales de Medecine, tome vi. "This gentleman," says our author, "lays it down as a principle, that, in the sound state, the mucous membrane of the stomach and bowels is white, or white with a slight tinge of rose. This assertion is true; but the question is, under what circumstances the membrane is white, or slightly rose-coloured? and this M. Rousseau could not determine in the conclusions drawn from his interesting observations. The recital of the observations themselves is sufficient to prove this. Thus, the three first individuals (who were executed), in whom he examined the digestive organs a few moments after death, showed the membrane white or whitish; neither the stomach nor intestines contained any aliments. In the second, it is true, the stomach had in it a portion of red wine; but it is not stated that any food was found along with this. In addition to the arguments used by the author of this memoir to prove that the whiteness was not the effect of the hemorrhage from the decapitation, may now be added the testimony of the observations proper to myself, and in which I found the membrane of the stomach of a white, more or less marked. In the three other individuals, the post-mortem examination of whose bodies is recorded by M. Rousseau, the stomach and commencement of the alimentary canal did contain food, and the mucous membrane in them was rose-coloured. In the fifth instance, in particular, it was observed that the mesenteric ganglia suffered a white fluid, having the characters of chyle, to exude on being cut; and, lastly, in the sixth observation, M. Heller found the stomach sound, its mucous membrane perfectly white; the small intestines contained a small quantity of food, and were reddish.

"These cases bear a strong analogy to mine, and M. Rousseau might have deduced from them similar conclusions; but he did not draw all the consequences which naturally flow from them. The author quoted appears to me to deny too strongly the passive nature of congestions of blood in the mucous membrane, in the case of affection of the heart or embarrassment of the circulation. If he had admitted, as a consequence of his observations, that the mucous membrane of the stomach and duodenum might become rose-coloured during digestion, he would not have been in haste to say that the subject of his seventh observation had an affection of the stomach, because the mucous membrane was found of a deeper rosecolour towards the lower part of the viscus, and appearing, after a few washings, covered over with small patches and bands. Nothing can be more unfounded than this opinion, particularly when we consider that the stomach of this individual contained wine and food imperfectly converted into chyme. I shall demonstrate in another place, that little patches and bands of a red colour on the mucous membrane, are not always the consequence of inflammation. The author never speaks of that slight ash-coloured shade which usually mingles with the white of the mucous membrane. I do not, therefore, admit his conclusions, except with certain restrictions, which may easily be perceived on comparing his inductions with mine."

*Of the glands of the mucous membrane of the alimentary canal.*—Some historical researches on the anatomy of these glands precede the description of M. Billard. He remarks, that they were noticed by T. Willis; that Stenon, Malpighi, Glisson, &c. have mentioned them in their works; but that it is to Peyer, who gave them their name, and Bruner, who followed him, that we owe our exact knowledge of these glands,—so exact, indeed, that little remains to be added to what they have said. They have been described with care by Beclard and Meckel; and it is from the materials furnished by these various sources, that M. Billard gives the following description:—

These glands ought to be studied in their healthy and diseased condition. They must not be confounded with the glands of Lieberkühn, the existence of which is doubted by Cuvier, and which Meckel does not appear to have seen in his microscopic observations. These glands are not scattered at hazard on the internal surface of the digestive canal; they observe an order which it is of importance to examine: thus they are, first, isolated or solitary; secondly, grouped in little irregular masses; thirdly, united into oval or olive-shaped patches. Those which are isolated offer the most simple state of glandular structure: they are principally found in the stomach, in the pyloric region, in the duodenum, in the cœcum, and the rest of the large intestines. They occupy indifferently the free or adherent surface of the intestine, the summits of the valvulæ, and their interstices. Their size, in the healthy condition, never exceeds that of a millet-seed; in their centre may be detected an almost imperceptible point, which indicates the orifice of their excretory duct; they are white, soft, and are broken down with the utmost facility. They may be named *criptæ muciparæ*. These cryptæ unite, and form little irregular masses, which are generally found at the adherent edge of the digestive tube. At the inferior third of the ileum are often found patches, the number of which varies, but the oblong form of which, and the situation on the free edge of the intestine, are constant. They are limited by a border, which is but little elevated, on the level of which the valvulæ conniventes are always interrupted. They present three degrees of development, at each of which they have a particular aspect:—First. They present a surface which is simply wrinkled or puckered; glandular granulations are not yet perceptible; they appear to be situated beneath the membrane, which they elevate a little: they form in this state what are called by the author *plaques gauffrees.* Secondly. In the midst of these wrinkled patches some white granulations, occasionally pointed at the summit, appear here and there; and between them may be perceived a mucous fold, which unites them to one another. Thirdly. The entire patch is studded with elevations, generally pointed at their summit, and which are interspersed with folds of the mucous membrane: sometimes the *ensemble* of these folds and glands gives them a certain degree of resemblance to a piece of coarsely polished granite. Such are the appearances of the glands of Peyer, in their sound state. They are not found in every subject; nevertheless they are very frequent. Their presence does not indicate a diseased state of the mucous membrane; but they are themselves susceptible of certain changes, which the author promises to explain in another place.

Some general conclusions follow, and terminate the Essay; but, as these are mere repetitions of what has already been stated in different parts of this analysis, any one who has read it must already have made them for himself; and those who might be desirous of jumping to the conclusions without perusing what precedes, could not possibly understand them.

---

